# Metagenomic next-generation sequencing in diagnosing rhino-orbital-cerebral mucormycosis presenting as cerebral Infarction: a case series and diagnostic analysis of seven patients

**DOI:** 10.3389/ffunb.2026.1751546

**Published:** 2026-01-22

**Authors:** Fei Yang, Chenglin Yang, Hongqiang Li, Xiaojuan Zhang, Xianfei Ding, Shuguang Zhang

**Affiliations:** 1 Critical Care Medicine, Department of Emergency Medicine, The First Affiliated Hospital of Zhengzhou University, Zhengzhou, Henan, China; 2 Department of Critical Care Medicine, The First Affiliated Hospital of Zhengzhou University, Zhengzhou, China; 3 The First Clinical Medical College of Zhengzhou University, Zhengzhou, China

**Keywords:** cerebral infarction, diagnosis, metagenomic next-generation sequencing, mucormycosis, rhino-orbital-cerebral mucormycosis

## Abstract

**Introduction:**

Rhino-orbital-cerebral mucormycosis (ROCM) is a rare, rapidly progressive, and fatal invasive fungal infection. This case series is the first to systematically characterize ROCM presenting primarily as cerebral infarction on imaging and highlights the value of metagenomic next-generation sequencing (mNGS) in the early diagnosis of such critical and atypical cases.

**Main symptoms and important clinical findings:**

All seven patients had diabetes mellitus, with six concurrently presenting with ketoacidosis. Universal clinical features included fever and a fixed, dilated pupil. Most patients exhibited facial swelling (6/7, 85.7%) and visual impairment (5/7, 71.4%). Cerebral infarction was confirmed by head magnetic resonance imaging (MRI) in all individuals.

**The main diagnoses, therapeutic interventions, and outcomes:**

The diagnosis was confirmed in all cases by the detection of Rhizopus species sequences via mNGS of cerebrospinal fluid (CSF). Six patients received treatment with amphotericin B cholesteryl sulfate complex, and two of these also underwent surgical debridement. Ultimately, only one patient survived, yielding a mortality rate of 85.7% (6/7).

**Conclusion:**

ROCM should be highly suspected in diabetic patients presenting with acute cerebral infarction accompanied by fever and facial or ocular symptoms. mNGS enables rapid and early etiological diagnosis of ROCM, which is crucial for improving outcomes. Earlier diagnosis, combined antifungal therapy, and surgical intervention may be associated with better prognosis.

## 1 Introduction

Rhino-orbital-cerebral mucormycosis (ROCM) is an acute, aggressive, and highly fatal invasive fungal infection (Saied et al., 2021). The causative agents, fungi of the order Mucorales, particularly the genus Rhizopus, are angioinvasive, frequently invading blood vessel walls leading to thrombosis and tissue necrosis, resulting in devastating clinical outcomes (Reid et al., 2020). ROCM remains relatively rare in clinical practice. Its manifestations are complex and non-specific, often leading to misdiagnosis, causing diagnostic delays and therapeutic failure (Steinbrink and Miceli, 2021; Niculae and Craciun, 2024). Diabetic patients, especially those with diabetic ketoacidosis (DKA), represent the highest risk group for ROCM (Chakrabarti et al., 2006; Wei et al., 2022). Fungal vasculitis secondary to cerebral infarction is a characteristic yet easily overlooked presentation (Meena et al., 2024). Conventional diagnostic methods, such as tissue culture and histopathology, remain the gold standard but suffer from low positivity rates, long turnaround times (Reid et al., 2020). Serological markers like (1,3)-β-D-glucan (G-test) and galactomannan (GM-test) are insensitive for mucormycosis (Roden et al., 2005; Rüping et al., 2010). mNGS has been widely applied in diagnosing challenging and critical infections (Zhao et al., 2021; Li et al., 2024; Zhao et al., 2024).In central nervous system (CNS) infections, mNGS can rapidly identify fastidious pathogens directly from cerebrospinal fluid (CSF) (Guo et al., 2022) and provide insights into the virulence and pathogenicity of Mucorales (Baldin et al., 2018). However, its systematic application and evaluation in ROCM presenting primarily as cerebral infarction have rarely been reported.

This study retrospectively analyzed seven ROCM patients whose initial MRI presentation was cerebral infarction. Our aims were to: (1) systematically describe the characteristics of this distinct clinical entity; (2) thoroughly investigate the critical role of mNGS in its early and accurate diagnosis; and (3) analyze treatment responses and prognosis to inform clinical management.

## 2 Materials and methods

### 2.1 Study design and participants

**Figure 1:**
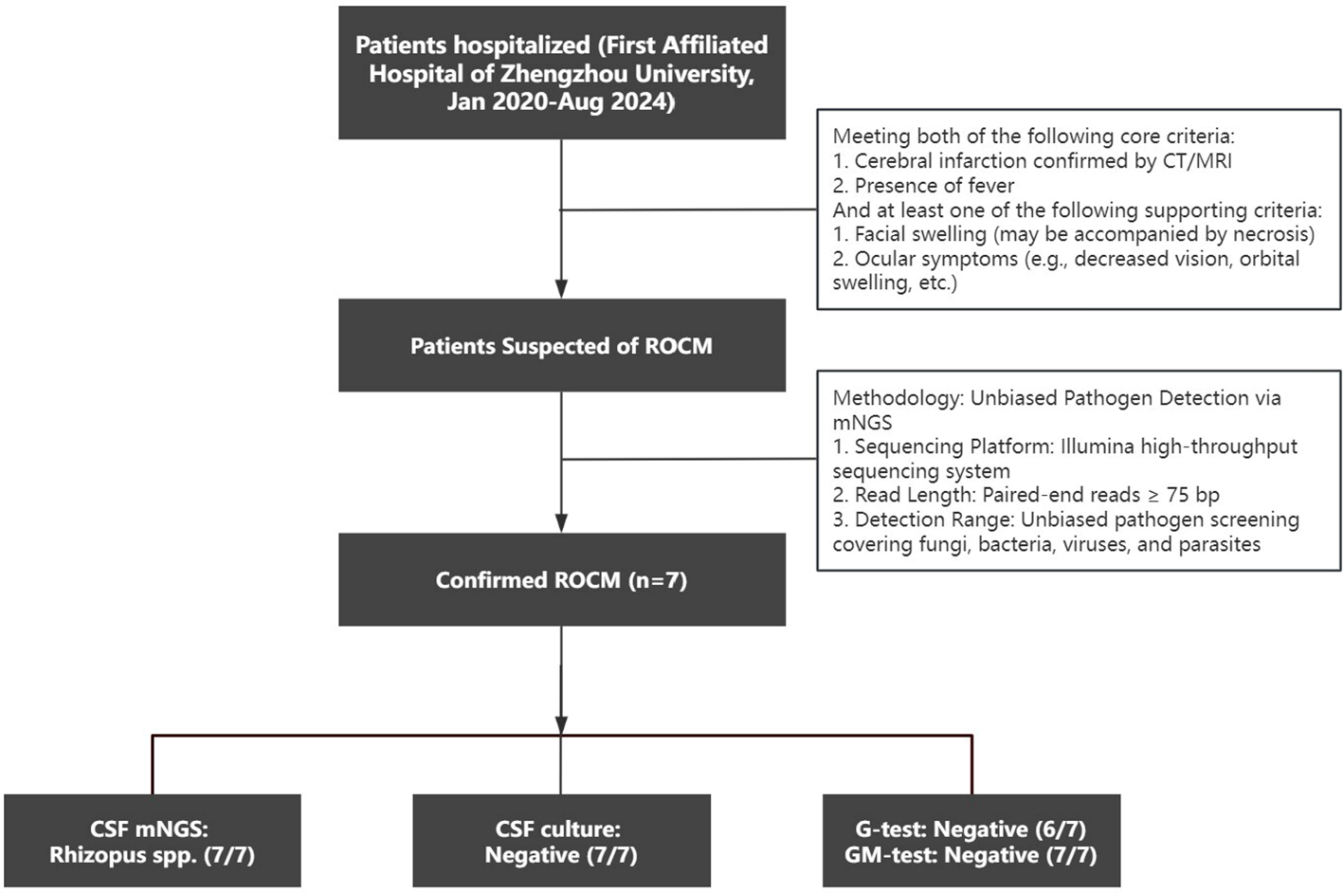
Flowchart of patient screening.

This study was a descriptive case series. We retrospectively included patients hospitalized at The First Affiliated Hospital of Zhengzhou University between January 2020 and August 2024, according to the following screening criteria: first, patients had to meet both of the following core criteria: ① cerebral infarction confirmed by CT or MRI, and ② the presence of fever. Additionally, at least one of the following supportive criteria was required: ① facial swelling (which could be accompanied by necrosis), or ② ocular symptoms (such as visual impairment, periorbital swelling, etc.). CSF mNGS was performed on patients who met the above criteria, and a total of 7 ROCM patients were ultimately diagnosed(**Figure 1**). The diagnosis of proven or probable ROCM followed the criteria outlined in the Global guideline for the diagnosis and management of mucormycosis (Cornely et al., 2019). The study was approved by the hospital’s Ethics Committee (Approval No: 2022-KY-1095-003), and informed consent was obtained from the patients or their families.

### 2.2 Data collection

Collected data included demographic information, underlying diseases, clinical manifestations, immunocompromised status (referencing host factors from the Revised Definitions of Invasive Fungal Disease (Donnelly et al., 2020)), laboratory results (complete blood count, inflammatory markers, glycated hemoglobin, CSF routine and biochemistry, and fungal-specific tests), imaging data (head MRI), treatment measures, and short-term outcomes.

### 2.3 Metagenomic next-generation sequencing

Total nucleic acid was extracted from the samples using a low-load nucleic acid extraction technique, targeting cell-free nucleic acids. High-throughput sequencing was performed on the Illumina platform with a read length of no less than 75 bp. Following quality control, the sequencing data were processed to efficiently deplete host-derived sequences using a constructed human pan-genome database. The remaining non-host sequences were then aligned against a pathogen pan-genome database for taxonomic identification and analysis of antibiotic resistance and virulence genes. Interpretation of pathogen detection results involved a comprehensive assessment based on both the “unique read count” (used for qualitative and relative quantitative analysis) and a multi-dimensional “confidence score” (evaluating identification reliability). This assay is an unbiased pathogen screening method with broad coverage, including but not limited to bacteria, fungi, viruses, and parasites.

### 2.4 Statistical analysis

Due to the small sample size, descriptive statistics were employed. Continuous variables are presented as median (range), and categorical variables as frequency (percentage).

## 3 Results

### 3.1 Baseline characteristics

All seven patients were farmers. The median age was 56 years (26-73), with one male and six females. All patients had diabetes mellitus, sinusitis and cerebral infarction. Six (85.7%) had diabetic ketoacidosis. The median APACHE II score on admission was 13 (11-30), and the median SOFA score was 3 (1-8).

**Figure 2:**
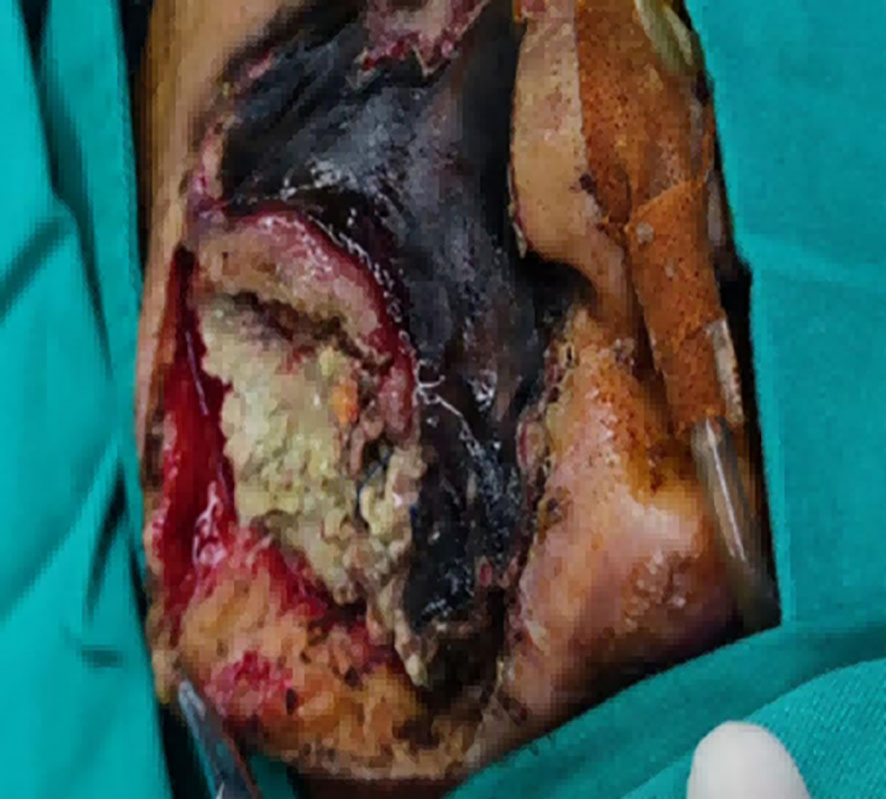
Black necrosis of maxillofacial region, the whole cheek fat infection can be seen during the operation, similar to mold bean residue, the maxilla involved black.

All patients presented with fever and a fixed, dilated pupil. Other common signs and symptoms included facial swelling (6/7, 85.7%), visual impairment (5/7, 71.4%), periorbital swelling (5/7, 71.4%), and facial necrosis (2/7, 28.6%). Physical examination revealed decreased visual acuity, a fixed and dilated pupil, and loss of both the pupillary light reflex and accommodation reflex on the affected side. (**Table 1** and **Figure 2**).

**Table 1:** Baseline characteristics of 7 cases with ROCM.

Item	Case 1	Case 2	Case 3	Case 4	Case 5	Case 6	Case 7
Sex	F	F	F	F	M	F	F
Age(years)	43	54	26	56	56	53	73
Occupation	farmer	farmer	farmer	farmer	farmer	farmer	farmer
Diabetes	+	+	+	+	+	+	+
Diabetic Ketoacidosis	+	+	+	+	+	+	–
Sinusitis	+	+	+	+	+	+	+
Cerebral infarction	+	+	+	+	+	+	+
Liver Function Tests	–	–	–	–	–	–	–
Renal Function Tests	–	–	–	–	–	–	–
Coagulation Panel	–	–	–	–	–	–	–
Cardiac Biomarkers	–	–	–	–	–	–	–
APACHE ;	13	12	11	17	12	30	21
SOFA	3	1	1	4	2	8	5
Peak Temperature/°C	37.9	39	38.7	38.7	38.4	38.2	39.2
Headache	–	–	–	+	–	–	–
Impaired Consciousness	–	–	–	–	–	–	–
Facial Swelling	–	+	+	+	+	+	+
Facial Necrosis	–	+	+	–	–	–	–
Visual Impairment	+	+	–	+	+	+	–
Periorbital Swelling	–	+	+	+	+	+	–
Dilated and fixed pupils	+	+	+	+	+	+	+
Chest Tightness	–	+	–	–	+	–	–
Abdominal Pain/Distension	+	–	–	–	–	–	–
Fatigue	+	+	–	–	+	–	+

F, Female; M, Male; +, Present; -, Absent.

### 3.2 Diagnosis

#### 3.2.1 Laboratory findings

Laboratory tests revealed significantly elevated inflammatory markers: median white blood cell count 22.2 × 10^9^/L (14.11-50.89), median procalcitonin 1.0 ng/mL (0.154-12), and median C-reactive protein 207.37 mg/L (137.89-337.34). Six (85.7%) patients had positive urine ketones with varying degrees of ketoacidosis. The median glycated hemoglobin was 12.3% (10.20-14.80). The positivity rates for G-test and GM-test were low (1/7 and 2/7, respectively). CSF analysis showed elevated pressure (>200 mmH_2_O in 5/7), mild to moderate pleocytosis (median 288 × 10^6^/L), and elevated protein levels (median 511 mg/L). (**Table 2**).

**Table 2:** Laboratory findings in seven patients with rhino-orbital-cerebral mucormycosis.

Item	Case 1	Case 2	Case 3	Case 4	Case 5	Case 6	Case 7
Complete Blood Count							
WBC (×10^9^/L)	50.89	22.2	26.17	20.2	23.37	14.11	18.63
Neutrophils (%)	91.9	89.8	94.3	91.3	88.7	80.5	93.7
Inflammatory Markers							
PCT(ng/mL)	12	1.42	1	0.324	1.152	0.154	0.44
CRP(mg/L)	337.34	137.89	207.37	207.55	141.34	176.39	219.03
IL-6 (pg/mL))	132.2	137.89	210.5	270	44.1	121	712.89
Urinalysis							
Ketones	3+	2+	1+	2+	1+	3+	–
Protein	1+	1+	1+	–	3+	1+	–
Glycemic Control							
HbA1c (%)	10.6	12.3	14.1	14.8	12.5	10.8	10.2
Cerebrospinal Fluid							
Pressure (mmH_2_O)	65	260	240	215	140	230	200
Nucleated cells (×10^2^/L)	65	352	173	288	330	854	30
Polymorphonuclear (%)	88.7	35.7	8.4	52.9	89	67.6	0.85
Chloride (mmol/L)	127	133	147	112	146.8	121.4	134.5
Glucose (mmol/L)	6.37	5.49	7.03	6.74	7.17	11.16	12.48
Total Protein (mg/L)	493	511	262	751	767.8	1256	303.9
Fungal Serology							
G-test (pg/mL)	<37.5	<37.5	<37.5	95.9	<37.5	<10	<37.5
GM-test (μg/L)	0.27	0.43	0.31	0.11	<0.25	0.173	1.43
CSF culture	–	–	–	–	–	–	–
mNGS	CSF: *R. oryzae* (874)	BALF: *R. oryzae* (54); CSF: *R. oryzae* (637)	CSF: *R. oryzae* (1115); Tissue: *R. oryzae* (4097)	CSF: *R. arrhizus* (1973)	Blood: *R. oryzae* (18); CSF: *R. oryzae* (1585)	CSF: *R. oryzae* (3613)	CSF: *R. delemar* (62), *R. oryzae* (8); BALF: *R. delemar* (544), *R. oryzae* (37)
Imaging Findings							
CT	Sinusitis	Sinusitis	Sinusitis	Sinusitis	Sinusitis	Sinusitis	Sinusitis
MRI (Non-contrast)	Sinus-Orbital Apex-Cavernous Sinus, Sinus-Frontal Lobe patchy, Iso-/Slightly hyperintense T1, Slightly hyperintense T2 signals	Sinus-Temporal Lobe-Basal Ganglia-Thalamus-Brachium Pontis involvement, Slightly hyperintense T1, Slightly hyperintense T2 signals	Maxillofacial-Orbital Apex-Retro-orbital-Cavernous Sinus, Right ICA involvement, Right Frontal Lobe, Basal Ganglia, Slightly hyperintense T1, Slightly hyperintense T2 signals	Left ICA involvement, Left Fronto-parietal Lobe patchy, Slightly hyperintense T1, Slightly hyperintense T2 signals	Sinus-Frontal Lobe, Bilateral Frontal Lobes, Right Parietal Lobe, Basal Ganglia, and Corpus Callosum patchy, Slightly hyperintense T1, Slightly hyperintense T2 signals	Sinus-Frontal Lobe-Basal Ganglia-Thalamus patchy, Slightly hyperintense T1, Slightly hyperintense T2 signals	Sinus-Right Frontal Lobe, Basal Ganglia patchy, Slightly hyperintense T1, Slightly hyperintense T2 signals
Contrast-enhanced MRI	No enhancement	Meningeal enhancement at temporal pole	Linear enhancement	Linear enhancement	\	\	\
DWI	High signal (Restricted diffusion)	High signal (Restricted diffusion)	High signal (Restricted diffusion)	High signal (Restricted diffusion)	High signal (Restricted diffusion)	High signal (Restricted diffusion)	High signal (Restricted diffusion)
Complications							
Shock	+	+	–	–	+	+	–
Respiratory Failure	+	+	–	+	+	+	+
Mechanical Ventilation	+	+	–	–	+	+	+
Treatment, and clinical outcomes							
Time to Diagnosis (days)	3	10	2	6	2	5	10
Antifungal Therapy	AmB-CS + POS	AmB-CS + POS	AmB-CS + POS	AmB-CS	AmB-CS	AmB-CS	None
Surgery	No	Yes	Yes	No	No	No	No
Length of Hospital Stay (days)	4	17	12	7	13	7	11
Outcome	Death	Death	Survived	Death	Death	Death	Discharged

mNGS, metagenomic next-generation sequencing; CSF, cerebrospinal fluid; BALF, bronchoalveolar lavage fluid; AmB-CS, amphotericin B cholesteryl sulfate complex; POS, posaconazole. + indicates presence, - indicates absence. The numbers in parentheses following the pathogen indicate the number of specific reads detected; CT, Computed Tomography; MRI, Magnetic Resonance Imaging; DWI, Diffusion-Weighted Imaging; ICA, Internal Carotid Artery.

#### 3.2.2 mNGS findings

CSF mNGS successfully detected Rhizopus species sequences in all seven patients, including R. oryzae, R. arrhizus, and R. delemar. In four patients, identical pathogen sequences were also detected in other samples (BALF, blood, or facial tissue), confirming the reliability of the mNGS results and suggesting disseminated infection. The median number of specific Rhizopus sequences in CSF was 874 (8-3613). (**Table 2**).

#### 3.2.3 Imaging findings

**Figure 3:**
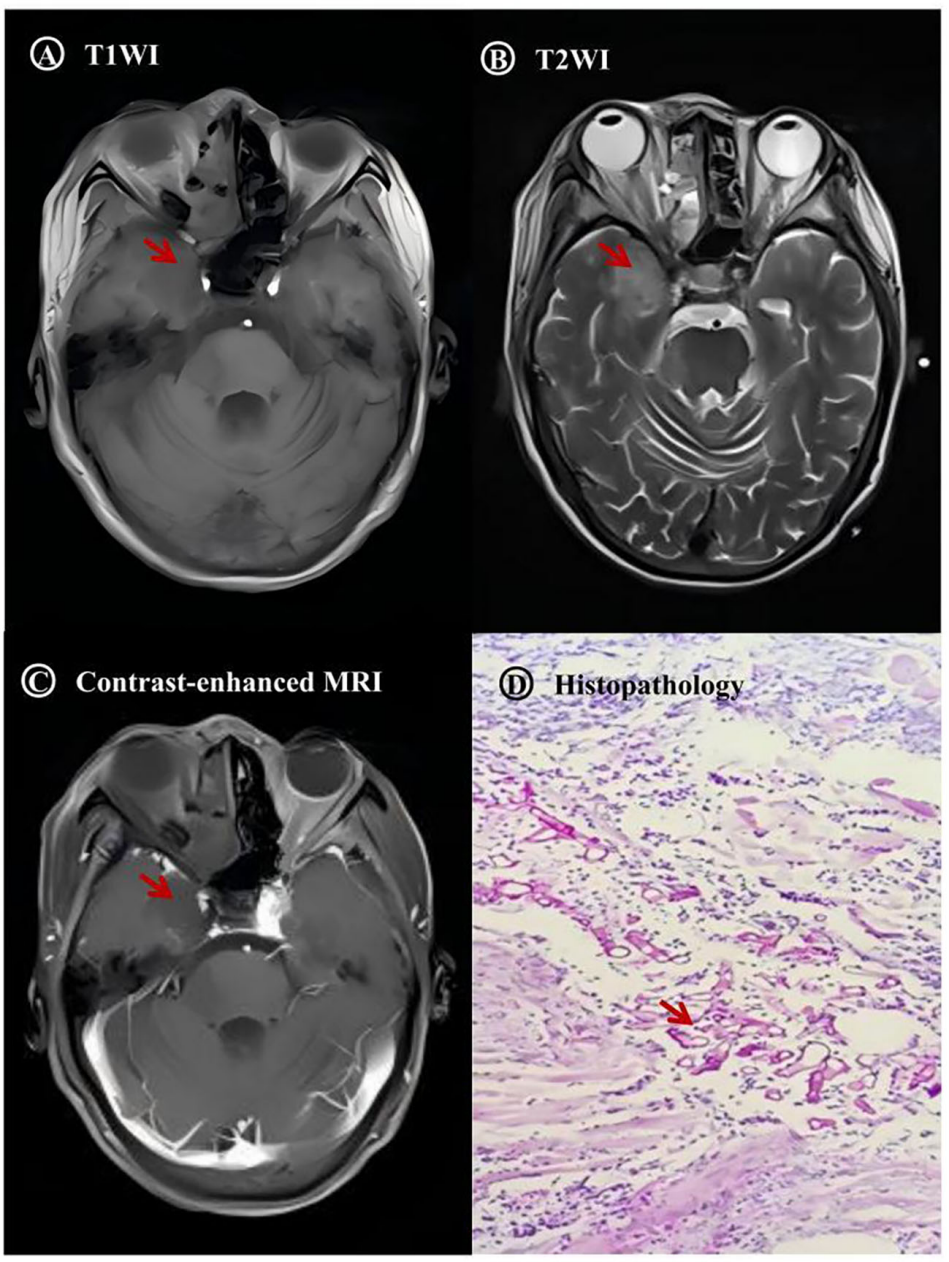
Case 2 MRI and histopathology. **(A–C)** Cerebral infarction **(D)** Mucorales.

All seven patients underwent both head computed tomography (CT) and magnetic resonance imaging (MRI). CT findings consistently indicated sinusitis in all cases. Head MRI revealed cerebral infarction foci in all patients, predominantly located in the frontal lobes and basal ganglia, corresponding to the supply territories of the affected internal carotid arteries. Contrast-enhanced MRI demonstrated abnormal enhancement and necrosis in the sinonasal cavity, orbital apex, and cavernous sinus regions. In some cases, characteristic signs of vascular invasion, specifically the “Black Turbinate sign”—manifested as non-enhancement in regions that normally enhance—were observed (**Table 2** and **Figure 3**).

#### 3.2.4 Diagnostic challenges

Initially, all cases in this series were primarily suspected to be cerebrovascular accidents due to their imaging presentation of cerebral infarction, posing a significant diagnostic challenge for ROCM. Conventional microbial cultures have low positivity rates, and invasive tissue biopsies carry high risks in critically ill patients. CSF cultures were negative. The G-test and GM-test offer limited diagnostic value for mucormycosis, easily leading to delays.

#### 3.2.5 Final diagnosis

The final diagnosis was Rhino-Orbital-Cerebral Mucormycosis (ROCM) with secondary fungal vasculitis and cerebral infarction.

### 3.3 Treatment and short-term outcomes

Six (85.7%) patients received treatment with amphotericin B cholesteryl sulfate complex (AmB-CS), three of whom were combined with posaconazole(POS). Two patients (28.6%, Cases 2 and 3) underwent surgical debridement. One patient (Case 7) was discharged without treatment.

Complications included respiratory failure (6/7, 85.7%, 5 requiring mechanical ventilation) and shock (4/7, 57.1%). Ultimately, only one patient (Case 3) survived, resulting in an overall mortality rate of 85.7%. The median time from symptom onset to diagnosis was 5 days (2–10 days). The sole survivor had the shortest time to diagnosis (2 days) and received early combined antifungal therapy and surgery. (**Table 2**).

### 3.4 Prognostic assessment

The overall prognosis of ROCM is extremely poor, particularly once intracranial dissemination occurs. In this series, the median time to diagnosis was 5 days, and the overall mortality rate was as high as 85.7%, indicating that early intervention is crucial.

## 4 Discussion

### 4.1 Clinical alert: cerebral infarction as a “warning sign” of ROCM

ROCM is relatively rare in clinical practice, typically presenting as sporadic cases with a fulminant and rapidly progressive course (Singh and Vishnu, 2021). The spores initially enter through the nasal cavity, causing necrosis and local symptoms such as nasal congestion and discharge. Subsequently, the infection spreads to the maxillary and ethmoid sinuses, orbit, cavernous sinus, meninges, and finally, the brain (Farmakiotis and Kontoyiannis, 2016). However, intracranial infection secondary to Mucorales lacks specific clinical manifestations and can closely mimic tuberculous meningitis, making it highly susceptible to misdiagnosis or missed diagnosis (Gavito-Higuera et al., 2016).

This study is the first to highlight the unique clinical phenomenon of cerebral infarction serving as the primary and core imaging manifestation of ROCM. All patients had comorbid diabetes with poor glycemic control (HbA1c >10%), and the majority presented with concurrent DKA, which is consistent with the typical predisposing background for mucormycosis (Prakash and Chakrabarti, 2019). Fever, acute facial swelling, rapid visual decline, and fixed dilated pupils constituted the typical clinical picture in this cohort. However, the most critical finding was that head MRI unequivocally demonstrated cerebral infarction in all cases, likely a result of fungal vascular invasion triggering inflammatory thrombosis and tissue necrosis (Vaughan et al., 2018). This finding carries significant warning significance for clinical practice.

### 4.2 Diagnostic challenges and technological innovation: from traditional methods to the application of mNGS

Imaging is one of the primary means for diagnosing ROCM. Contrast-enhanced MRI can clearly display the extent of necrosis in involved structures and the invasion of surrounding tissues (Sreshta et al., 2021). The “Black Turbinate sign” appears on T1WI, corresponding to a necrotic eschar observed on nasal endoscopy. Early recognition of this sign can facilitate the early diagnosis of ROCM (Safder et al., 2010), but it cannot achieve etiological confirmation by itself.

Histopathology remains the gold standard for definitive diagnosis (Pai et al., 2021). On pathological sections, Mucorales hyphae exhibit broad (6–25 μm), pauciseptate, branching (at 90° angles) morphology (Reid et al., 2020). However, obtaining viable intracranial pathological tissue carries high risks. If the disease is suspected, timely endoscopic sinus surgery should be performed to obtain sinus tissue for pathological examination.

Culture and the G/GM tests are methods for identifying fungal infections, yet their results are often unsatisfactory. In this study, CSF cultures were negative in all cases, and only a few patients tested positive for G/GM tests, which was considered possibly due to concurrent Aspergillus infection, consistent with previous reports (Rüping et al., 2010; Chinese Society of Neuroinfectious Diseases and Cerebrospinal Fluid Cytology and Neuroinfectious Diseases Committee of Neurologist Branch of Chinese Medical Doctor Association, 2023). Furthermore, while the epidemiological background, clinical manifestations, and imaging features in our case series were largely consistent with those reported previously, the time to diagnosis was significantly shorter than in earlier studies (Onyi et al., 2022; Sweet et al., 2023; Aicher et al., 2024). This difference primarily stemmed from the early and systematic application of mNGS for etiological screening in our cohort (Zhang et al., 2022). Our results strongly support the use of mNGS as a first-line diagnostic tool for suspected central nervous system ROCM, especially when conventional methods are negative and the patient is critically ill.

### 4.3 Treatment strategies and prognostic analysis

Current management of ROCM emphasizes multidisciplinary collaboration, focusing on early diagnosis, timely antifungal therapy, aggressive surgical debridement, and control of underlying diseases. AmB-CS are recommended as the first-line drugs for mucormycosis involving the central nervous system due to their ability to achieve relatively high concentrations in the CNS (Groll et al., 2000; Cornely et al., 2019). Isavuconazole and POS, available as oral or intravenous formulations, are recommended as second-line agents or for step-down maintenance therapy (Czech and Cuellar-Rodriguez, 2025). If the patient has an immunodeficiency, the duration of antifungal therapy is recommended to be extended until the signs and symptoms have resolved (Niculae and Craciun, 2024).

Mucorales can cause intravascular thrombosis, and the risk of hemorrhage is considered low (Sun et al., 2009). In this study, Case 2 died postoperatively due to secondary cerebral hemorrhage and brain herniation, suggesting that surgery itself may pose a fatal risk for critically ill patients who have already developed extensive intracranial vascular invasion. Case 3 survived but was left with severe maxillofacial disfigurement. This underscores that the necessity and timing of surgery for patients with multi-system involvement and poor general condition remain controversial and require individualized assessment.

Although the small sample size of this study precludes statistical comparison, a descriptive comparison between the sole survivor (Case 3) and the deceased cases still provides important insights. The surviving patient had a shorter time from symptom onset to diagnosis (2 days) and received an early, combined treatment regimen (initiation of AmB-CS with POS upon diagnosis, and surgical debridement during hospitalization). Furthermore, the survivor’s APACHE II score (Zhao et al., 2021) and SOFA score (Saied et al., 2021) were at the lower end of the median and range observed in the deceased cases (APACHE II: 17 [12-30]; SOFA: 4 [1-8]). These observations collectively suggest that for such critically ill patients, lower initial disease severity, minimizing the time to diagnosis, and promptly initiating aggressive comprehensive treatment, including surgery, may be associated with improved outcomes.

### 4.4 Analysis of the clinical utility of mNGS

mNGS enables rapid and broad-spectrum detection of pathogens. However, its widespread clinical adoption remains constrained by factors such as high cost, stringent technical and equipment requirements, and limited accessibility in primary healthcare settings. Nevertheless, in cases of severe infection, when conventional tests yield negative results, or in immunocompromised hosts with complex or rare infections, the diagnostic value of mNGS often outweighs its economic cost, offering significant clinical benefits. With ongoing technological advancements, gradual reduction in costs, and further optimization of workflows, mNGS is expected to see broader application in frontline clinical practice in the future.

## 5 Limitations

This study has several limitations, primarily its small sample size and single-center, retrospective design, which preclude robust statistical analysis. mNGS itself has challenges, including cost, the potential detection of colonizers, and the need for specialized bioinformatics expertise. Future larger-scale, multi-center prospective studies are needed to validate our findings and explore potential correlations between mNGS read counts and disease severity or prognosis.

## 6 Patient perspective

Due to the retrospective nature of this study and the unfortunate demise of the majority of patients, we were unable to systematically collect the patients’ own perspectives on their treatment experiences.ROCM should be highly suspected in diabetic patients presenting with acute cerebral infarction accompanied by fever and facial or ocular symptoms. mNGS enables rapid and early etiological diagnosis of ROCM, which is crucial for improving outcomes. Earlier diagnosis, combined antifungal therapy, and surgical intervention may be associated with better prognosis.

## Data Availability

The original contributions presented in the study are included in the article/Supplementary Material. Further inquiries can be directed to the corresponding author.
